# A paper biosensor for overcoming matrix effects interfering with the detection of sputum pyocyanin with competitive immunoassays

**DOI:** 10.1007/s00604-023-06017-1

**Published:** 2023-10-16

**Authors:** Cristina Adrover-Jaume, Antonio Clemente, Bárbara Rodríguez-Urretavizcaya, Lluïsa Vilaplana, M. Pilar Marco, Estrella Rojo-Molinero, Antonio Oliver, Roberto de la Rica

**Affiliations:** 1 Multidisciplinary Sepsis Group, Hospital Universitario Son Espases, Health Research Institute of Balearic Islands (IdISBa), Palma, Spain; 2 Department of Chemistry, University of the Balearic Islands, Palma, Spain; 3 Nanobiotechnology for Diagnostics (Nb4D), Department of Surfactants and Nanobiotechnology, Institute for Advanced Chemistry of Catalonia (IQAC), Barcelona, Spain; 4 CIBER de Bioingeniería, Biomateriales y Nanomedicina (CIBER-BBN), Barcelona, Spain; 5 Microbiology Department, Hospital Universitario Son Espases, Health Research Institute of Balearic Islands (IdISBa), Palma, Spain; 6 CIBER de Enfermedades Infecciosas (CIBERINFEC), Instituto de Salud Carlos III, Madrid, Spain

**Keywords:** Pyocyanin, *Pseudomonas*, Sputum, Biosensor; Au nanoparticles, Paper-based

## Abstract

Detecting sputum pyocyanin (PYO) with a competitive immunoassay is a promising approach for diagnosing *Pseudomonas aeruginosa* respiratory infections. However, it is not possible to perform a negative control to evaluate matrix-effects in competitive immunoassays, and the highly complex sputum matrix often interferes with target detection. Here, we show that these issues are alleviated by performing competitive immunoassays with a paper biosensor. The biosensing platform consists of a paper reservoir, which contains antibody-coated gold nanoparticles, and a substrate containing a competing recognition element, which is a piece of paper modified with an albumin-antigen conjugate. Detection of PYO with a limit of detection of 4.7·10^−3^ µM and a dynamic range between 4.7·10^−1^ µM and 47.6 µM is accomplished by adding the sample to the substrate with the competing element and pressing the reservoir against it for 5 min. When tested with patient samples, the biosensor was able to qualitatively differentiate spiked from non-spiked samples, whereas ELISA did not show a clear cut-off between them. Furthermore, the relative standard deviation was lower when determining sputum with the paper-based biosensor. These features, along with a mild liquefaction step that circumvents the use of harsh chemicals or instruments, make our biosensor a good candidate for diagnosing *Pseudomonas* infections at the bedside through the detection of sputum PYO.

**Graphical Abstract:**

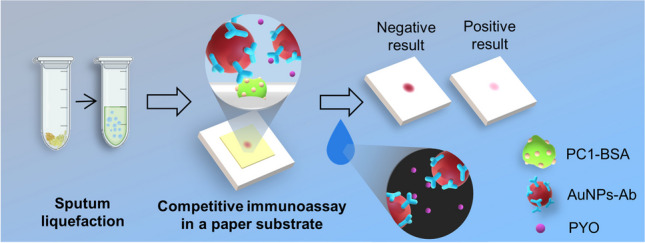

**Supplementary Information:**

The online version contains supplementary material available at 10.1007/s00604-023-06017-1.

## Introduction

*Pseudomonas aeruginosa* is a common opportunistic pathogen in patients who are immunocompromised either due to a medical condition (e.g., malignancy, autoimmune and inflammatory diseases) or immunosuppressive therapies (e.g., medications for organ transplant patients) [1, 2]. Indeed, it is one of the main causes of hospital-acquired pneumonia especially among mechanically ventilated patients in intensive care units [3, 4]. Nosocomial strains are multidrug resistant, making this pathogen a life-threatening agent. This means that antibiotic coverage must be carefully designed taking into consideration the few options still available to eliminate *P. aeruginosa* [5, 6]. Bacterial culture of respiratory samples is the gold standard test for detecting pathogens causing pneumonia but this method demands several days for a result and, meanwhile, if *P. aeruginosa* infection is mismanaged by the empirical therapy, the patients’ illness can rapidly progress towards deadly sepsis [7, 8]. Thus, there is a specific need to develop technologies for the rapid detection of *P. aeruginosa* in respiratory samples at the bedside so that antibiotic therapies can be fine-tuned according to the presence of this multidrug-resistant pathogen.

Pyocyanin (PYO) is a small toxin (≈210 Dalton) exclusively produced and secreted by *P. aeruginosa* [9]. Therefore, detecting PYO in respiratory samples, within minutes and at the bedside, could reveal the presence of *Pseudomonas* and help personalize antibiotic options. Recent works have reported several approaches for detecting PYO, including electrochemical sensors based on paper, gold nanoparticles (AuNPs), or carbon quantum dots [10–14], ELISA [15, 16], surface-enhanced Raman spectroscopy (SERS) [17–19], and heat-transfer methods [20]. Some approaches used antibodies for the specific recognition of PYO, whereas others relied on the electrochemical properties or spectral fingerprint of the molecule to detect it. Among these, antibody-based methods stand out for their high specificity, which is important when analyzing complex samples such as respiratory specimens. Indeed, detecting PYO in respiratory samples such as sputum and bronchial aspirate is challenging due to the sample matrix, which is made of highly cross-linked mucins with a heterogeneous and highly viscous, even semi-solid consistency [21]. This generates interferences or matrix effects that increase the intra- and inter-sample variability unless samples are properly processed. Furthermore, the detection of PYO in respiratory samples requires using a competitive immunoassay format because the molecule has a single epitope. This means that it is not possible to perform a negative control in order to evaluate and subtract matrix effects.

**Fig. 1:**
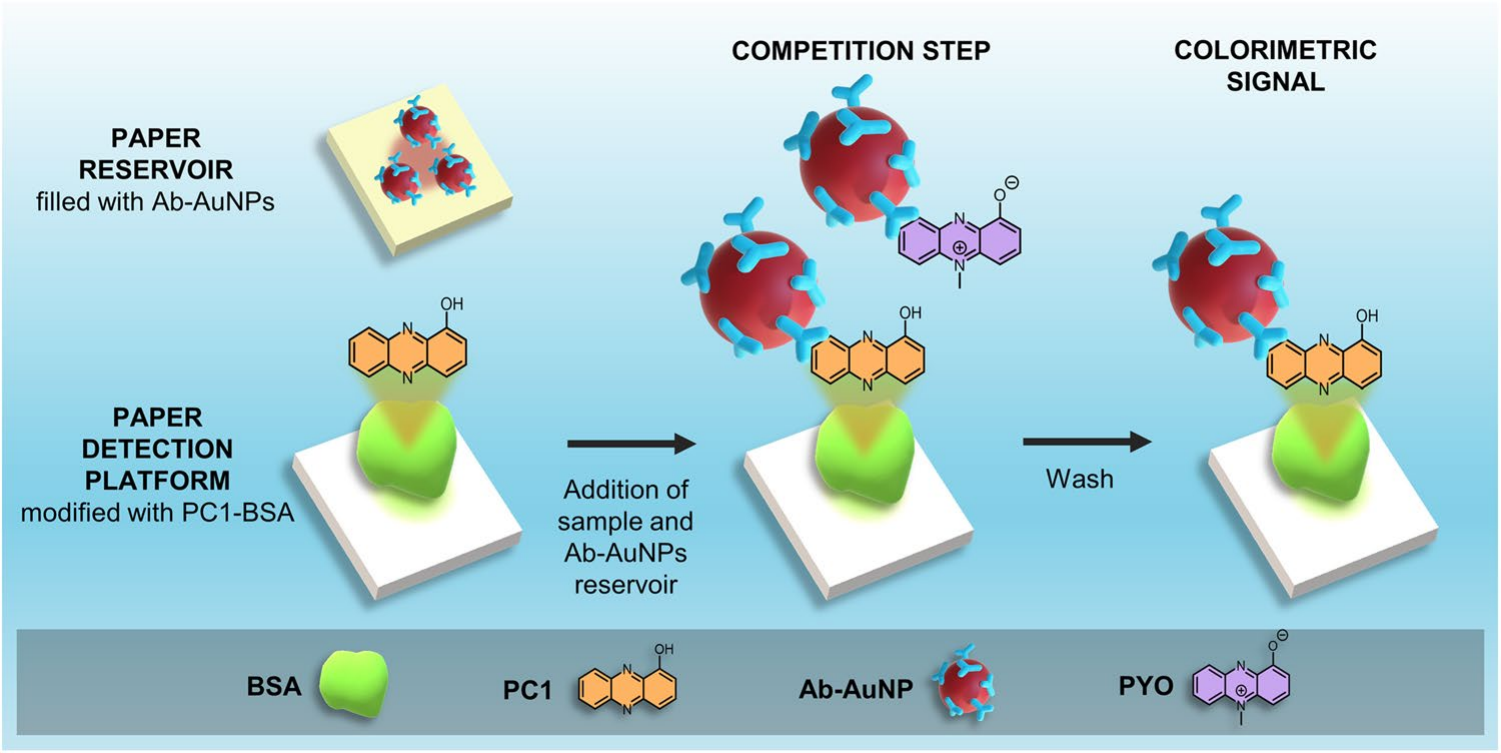
Schematic representation of the paper-based competitive immunoassay for detecting sputum pyocyanin. The biosensor consists of a paper strip with immobilized PC1-BSA (*1-hydroxyphenazine* conjugated to bovine serum albumin) as the competing recognition element (detection platform) and a paper-based reservoir of Ab-AuNPs. PC1 is the main metabolite of PYO synthesis pathway and can be recognized by the antibody. After an enzymatic liquefaction, the sample is added to the detection platform and then the reservoir of Ab-AuNPs is pressed against it for 5 min. During this step, the Ab-AuNPs are transferred from the reservoir and interact at the same time with the free PYO from the sample as well as with the paper-bound PC1-BSA (competition step). After washing, a colored spot remains on the paper whose intensity is inversely proportional to the concentration of PYO (colorimetric signal)

In this article, we introduce a paper biosensor for detecting sputum PYO within minutes and at the bedside that also reduces matrix effects hindering the detection of this molecule with traditional competitive ELISA (Fig. 1). To detect PYO, the sputum sample is first liquefied using an enzymatic method [22–24]. Previous approaches relied on using lengthy procedures involving organic solvents or strong acids and bases for PYO extraction that are difficult to implement at the bedside [15, 17]. By contrast, the enzymatic method only requires adding hydrogen peroxide for 1 min to mechanically disrupt the sample through the production of bubbles, in one step and without using instrumentation [22]. The biosensor consists of a paper substrate containing a competing recognition element, which has been modified with an albumin-antigen conjugate (PC1-BSA), and a reservoir, which contains 20 nm gold nanoparticles modified with anti-PYO mAb (mAb122) (Ab-AuNPs in Fig. 1). In competitive formats, 20 nm AuNPs are preferred over larger particles because the latter are coated with a larger number of antibodies, which may bind to many free PYO molecules and decrease the efficiency of the competition with paper-bound antigens. To detect PYO, the liquefied sample is added to the detection platform (a piece of paper containing the competing element PC1-BSA in Fig. 1) and subsequently the reservoir is pressed against it so that Ab-AuNPs are transferred. During this 5-min-incubation step, the Ab-AuNPs may simultaneously interact with the PYO released from the sample matrix and with the paper-bound PC1-BSA (competition step in Fig. 1). After washing, a colored spot remains on the paper whose pixel intensity is inversely proportional to the concentration of PYO in the sample (colorimetric signal in Fig. 1). It will be shown that it is possible to detect PYO in sputum samples within 6 min using this approach, whereas the detection with the gold standard competitive ELISA required 2 h and could not clearly identify PYO in all the specimens due to matrix effects. Supplementary Table S1 shows detailed comparison between this work and other reported methods for the rapid detection of *Pseudomonas* infections. Our results pave the way for detecting respiratory infections caused by *P. aeruginosa* with paper biosensors rapidly and at any point of health care, which is important for providing life-saving antibiotics in a timely manner.

## Experimental section

### Materials

Materials include the following: Whatman filter paper grade #41 and grade #1 (GE Healthcare Life Sciences), gold (III) chloride hydrate (99.9%, Sigma-Aldrich), sodium citrate tribasic dihydrate (99%, Sigma-Aldrich), 30% poly(sodium 4-styrenesulfonate) solution (PSS, Sigma-Aldrich), bovine serum albumin (BSA ≥ 98%, VWR Chemicals), sucrose (99.5%, Sigma-Aldrich), Tween 20 (Sigma-Aldrich), 6-[Fluorescein-5(6)-carboxamido] hexanoic acid N-hydroxysuccinimide ester (NHS-FL 75%, Sigma-Aldrich), pyocyanin (98%, Sigma-Aldrich), high affinity mouse monoclonal IgG antibodies anti-pyocyanin (mAb122) produced in-house [16], PC1-BSA bioconjugate (produced in-house, hapten density = 10), goat anti-mouse-IgG/HRP (Dako Agilent), sodium bicarbonate (99.7%, Sigma-Aldrich), sodium carbonate (≥ 97%, VWR Chemicals), 30% hydrogen peroxide solution (Sigma-Aldrich), 3,3′,5,5′-tetramethylbenzidine (TMB, 99%, Thermo Fisher), sulfuric acid (95%, VWR Chemicals), Bradford reagent (VWR Chemicals), PD-10 Desalting Column (GE Healthcare), 96-well maxisorp ELISA microplates (Thermo Scientific). PBS refers to phosphate buffered saline (pH 7.4), PBST refers to PBS with 0.1% Tween 20, and PBS-BSA refers to PBS containing 5 mg·mL^−1^ BSA).

### AuNPs modification with mAbs against pyocyanin

Twenty-nanometer AuNPs were synthesized using the Turkevich method [25]. In brief, 4 mM trisodium citrate was added to a boiling aqueous solution of 0.25 mM gold chloride for 15 min while vigorously stirring (final volume 250 mL). The resulting AuNPs were let to cool down at room temperature. The size and shape of freshly synthesized AuNPs were evaluated by TEM analysis using Talos TEM microscope operating at 80 kv. AuNPs were subsequently modified with specific mouse monoclonal IgG antibodies that recognize specifically PYO (mAb122). These high affinity antibodies have been raised using hapten PC1 (a 1:1 mixture of 9-hydroxy- and 6-hydroxy-phenazine-2-carobxylic acids), designed to recognize 1-hydroxyphenazine (PC1), which is the main metabolite of PYO synthesis pathway. We have previously demonstrated that the antibody can detect both PC1 and PYO [16].

We followed a physical adsorption protocol adapted from the literature in order to decorate AuNPs with mAb122 [26]. Briefly, 100 µL mAbs solution (156 µg·mL^−1^, in distilled water) was added to 1 mL AuNPs (previously adjusted at A_520_ = 0.6) and incubated for 20 min under vigorous stirring. Then, Ab-AuNPs were blocked by adding 100 μL BSA solution (10 mg·mL^−1^ in distilled water), under stirring for 20 min. Finally, the blocked Ab-AuNPs were centrifuged at 7500 r.p.m. for 12 min and the pellet was resuspended with 25 µL sucrose-BSA solution (50 mg·mL^−1^ and 1 mg·mL^−^, respectively, in distilled water). The final colloid was stored at 4 °C until use. Nanoparticle size was studied using nanoparticle tracking analysis (NTA) performed with a NanoSight NS 300 instrument. Measurements of ζ potential were made with a Malvern Zetasizer Nano-ZS90.

### Pyocyanin paper biosensor manufacturing

Paper biosensors were made of Whatman #41 paper sheets cut into 2 × 8 cm strips. The strips were subdivided into four 2 × 2 cm squares and folded like an accordion as shown in Fig. 1B. Then, the area containing the competing recognition element (the first square of the paper strip) was coated with 10 µL PC1-BSA bioconjugate solution (at 1.52 µM PC1) as competitive antigen. PC1-BSA was synthesized as previously described [15].

Ab-AuNPs reservoirs were made of filter paper sheets infused with polystyrene sulfonate (PSS) following our already published method [27]. Briefly, Whatman filter paper #1 was cut into 7 × 7 cm^2^ squares. Then, 3 mL 18% PSS was added to a 9 × 9 cm^2^ square glass recipient and the piece of paper was placed above the polymer solution. Next, the recipient was incubated at 37 °C for 30 min. Finally, reservoirs containing Ab-AuNPs were made by cutting the resulting PSS-infused paper into 0.5 × 0.5 cm^2^ squares, adding 0.5 μL Ab-AuNPs and letting it dry at room temperature (RT) for 10 min.

### Fluorescent labeling of BSA and imaging

BSA was labeled with amine-reactive fluorescein (NHS-FL) as follows. A total of 1 mg·mL^−1^ NHS-FL was added to 10 mg·mL^−1^ BSA in PBS for 30 min at RT. Then, the fluorescein labeled BSA (BSA-FL) was separated from unreacted NHS-FL using a PD-10 Desalting Column. Consequently, we spotted 10 µL BSA-FL (≈350 µg·mL^−1^) on Whatman #41 paper substrates and left them to dry at RT for 15 min. Next, the fluorescence was measured with a Typhoon FLA 9500 laser scanner (General Electric) by using the blue LD laser (473 nm) in the instrument. Then, we simulated the sample addition step during the competitive assay by rehydrating paper substrates with 0.5-mL distilled water. Finally, paper substrates were left to dry and scanned again. The fluorescent signal S was measured by densitometric analysis of the obtained images as described below.

### Pyocyanin detection by a paper-based direct competitive assay

Detection of PYO with the proposed biosensor proceeded as follows: First, 0.5 mL of PYO standard solutions (0, 4.76·10^−3^, 4.76·10^−2^, 4.76·10^−1^, 4.76, 47.6 µM, in PBS-BSA) or liquefied sputum samples was added to the area containing the competing recognition element of the folded biosensor (competing element PC1-BSA in Fig. 1). Immediately afterwards, Ab-AuNPs were transferred by pressing the reservoir against the paper biosensor for 5 min with a clamp. During this step, free PYO from the sample and paper-bound PC1-BSA bioconjugate compete to bind to Ab-AuNPs (competition step in Fig. 1). Finally, the reservoir was peeled off and the biosensor was washed with a total volume of 1 mL PBST dispensed in 6 sequential additions. The signals yielded by the biosensor were inversely proportional to the concentration of PYO within the sample. Paper substrates were scanned with an MFC-1910W scanner-printer (Brother), and the colorimetric signal *S* was measured within the obtained images by densitometric analysis as described below.

### Densitometry analysis

The fluorescent signals from BSA-F experiments and the colorimetric signals from PYO biosensor assays were converted to images as described above and measured by densitometry. Briefly, the pixel intensity (PI) in the grayscale channel of the signals was obtained by using the histogram function of the ImageJ software after selecting the region of interest (ROI). In grayscale, pure black and white colors yield PI values of 0 and 255, respectively. The signals *S* were obtained after subtracting the obtained PI value from the background, which yielded inverted signals compared to the raw data.

### Pyocyanin indirect competitive ELISA

The calibration plot for the detection of PYO with the standard indirect competitive ELISA method was performed as previously described [16]. Briefly, the 96-well ELISA plate was coated with 100 µL PC1-BSA bioconjugate (at 0.03 µM PC1) in coating buffer (0.05 M carbonate buffer, pH 9.6) overnight at 4 °C. Next, wells were washed 4 times with 0.05% PBST. Then, 50 µL of different PYO serial concentrations (0, 1.6·10^−4^, 1.6·10^−3^, 5.0·10^−3^, 2.0·10^−2^, 4.0·10^−2^, 2.0·10^−1^, and 1.0 µM in PBST) was added by triplicate to coated wells, and immediately afterwards, 50 µL mouse anti-PYO (mAb122) at 0.05 µg·mL^−1^ was added. After 30 min incubation at RT with gently shaking, wells were washed 4 times with 0.05% PBST and 100 µL anti-mouse-IgG/HRP at 0.5 µg·mL^−1^ was added for 30 min at RT without shaking. Then, wells were washed 4 times with PBST and 100 µL substrate solution (0.04 M sodium citrate buffer pH 5.5 + 0.4 mM TMB + 1.2 mM H_2_O_2_) was added for 15 min at RT. Finally, the reaction was stopped by adding 50 µL 4 N sulfuric acid and the absorbance at 450 nm was measured using a PowerWave HT microplate reader (Biotek).

### Sputum samples

Sputum samples were collected by the Department of Microbiology at Son Espases University Hospital (Balearic Islands). Quantitative culture test for bacterial pathogens was carried out by plate counting of bacterial colonies after seeding serial dilutions of sputum. Sputum samples containing a mixed flora or bacterial pathogens causing infection (bacterial load ≥ 10^5^ colony-forming units) different from *P. aeruginosa* were selected. Results of the bacterial culture test from samples included in this study can be found in supplementary Table S2. Raw sputum samples were kept at − 20 °C until used.

Prior to analysis, sputum samples were enzymatically liquefied as previously described [22]. Briefly, samples were thawed and 50–100 mg was weighed in a conical 15-mL polypropylene tube. Then, 0.3 M H_2_O_2_ in PBS was added at a 20:1 constant ratio (v/w) for 1 min at RT, which liquefies samples due to the oxygen bubbles generated by endogenous catalase in sputum.

### Matrix-effect studies

Interferences produced by sputum matrices were studied for both competitive assays (ELISA and the proposed paper-based biosensor). For this purpose, after liquefying sputum samples, we performed 1:20 and 1:50 dilutions in PBS-BSA for the biosensor or PBST for ELISA (hereinafter referred to as PBS) and, subsequently, non-diluted and diluted samples were spiked with a constant PYO concentration within the linear range of detection for each method (2.4·10^−2^ µM for the ELISA and 2.4 µM for the biosensor). Then, PYO detection was conducted by following the protocols detailed above. Non-spiked samples were analyzed in parallel as controls. Percentage of matrix effect was calculated as follows: matrix effect (%) = [(*S*_PBS_ – *S*_sputum_)/*S*_PBS_] × 100, where *S*_PBS_ are the blank *A*_450_ or *S* colorimetric signals produced in ideal conditions (with no matrix), and *S*_matrix_ are signals obtained when measuring non-spiked sputum samples._._ The accuracy of detection of spiked PYO in sputum samples was calculated as the relative error of the signal decrease due to PYO spiked into sputum with respect to that in PBS. Thus, considering the relative error as the quotient of the absolute error and the experimental value and being the absolute error the difference between the expected and the experimental value, then the relative error (%) = [(Δ_PBS_ – Δ_sputum_)/Δ_PBS_] × 100, where Δ_PBS_ is the maximal signal decrease due to PYO spike in PBS (with no matrix) with respect to those without PYO, and Δ_sputum_ is the signal decrease due to PYO spike in sputum with respect to the non-spiked sample.

## Results and discussion

**Fig. 2:**
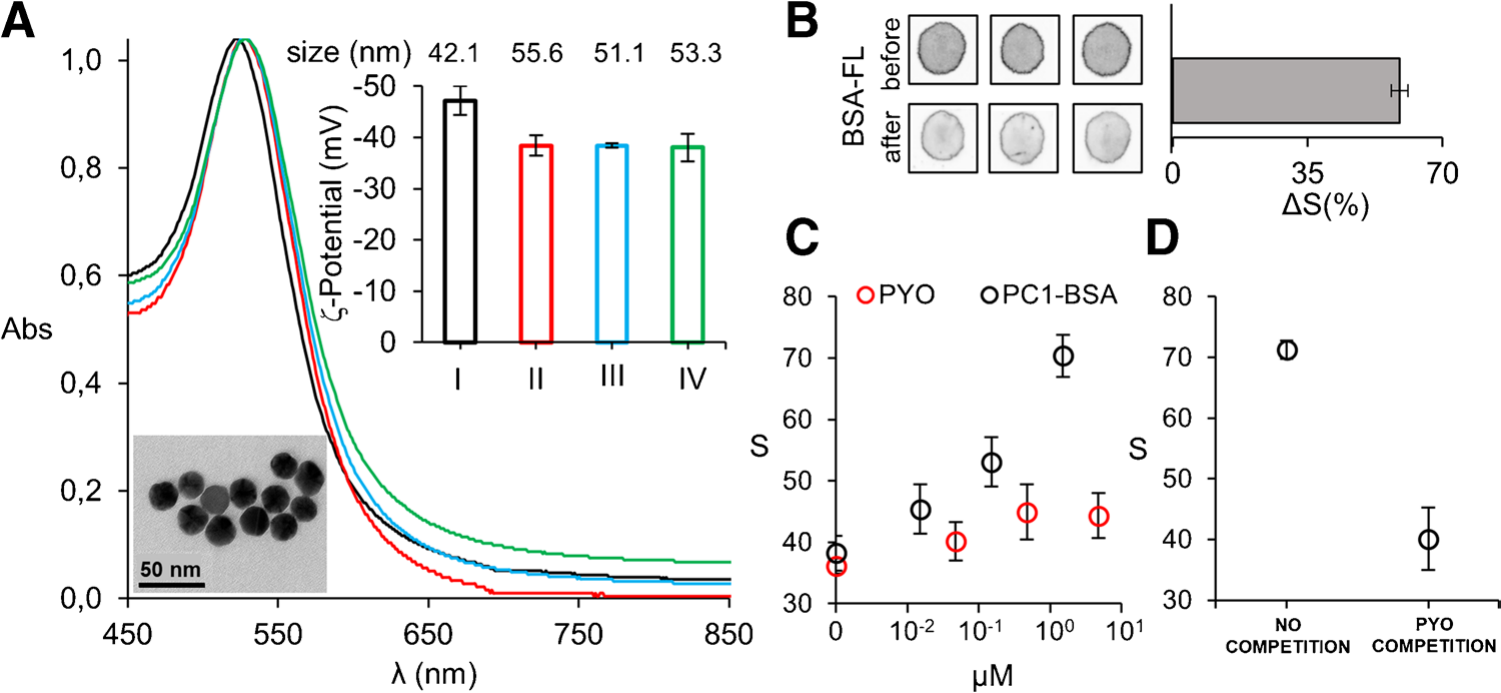
Biosensor characterization. **A** Vis–NIR spectroscopy of gold nanoparticles (AuNPs), hydrodynamic particle diameter determined by nanoparticle tracking analysis (size data in Inset) and ζ-potential measurements (bars in Inset) after the synthesis (I, black) and after each step of the functionalization protocol; addition of antibodies (II, red), stabilization with BSA (III, blue), and addition of sucrose (IV, green). Error bars are the standard deviation (SD) of 3 consecutive measurements. The inset picture in (**A**) represents a TEM image of freshly synthesized AuNPs. **B** Fluorescence images before and after rehydrating filter paper modified with fluorescein-BSA; Bars represent the percentage of fluorescence retained on the paper substrates (%Δ*S* = [(*S*_before_ − *S*_after_)/*S*_before_] × 100). Error bars are the SD of 3 independent measurements. **C** Colorimetric S signal produced by Ab-AuNPs during the direct detection of unconjugated pyocyanin (PYO, red dots) or BSA-conjugated antigen (PC1-BSA, black dots) physically adsorbed to paper substrates. Error bars are the SD of 3 independent measurements. **D** Colorimetric S signals produced by Ab-AuNPs in a direct paper-based competitive immunoassay in the absence of PYO (no competition) and with 47.6 µM PYO in PBS-BSA (PYO competition). Error bars are the SD of 3 independent measurements

Figure 2 shows experiments performed in order to characterize the different elements of the biosensor. Figure 2A shows characterization experiments for AuNPs performed at each step of the modification protocol with anti-PYO. In this Figure, TEM imaging of freshly synthesized AuNPs demonstrates a well-defined spherical shape (Fig. 2A, inset). The mean diameter of AuNPs calculated from TEM images was 21.2 ± 2.7 nm (supplementary Table S3). The LSPR in Fig. 2A shifts from 522 to 530 nm, the ζ-potential increases (from − 47.2 to − 38.0 mV), and the hydrodynamic diameter increases from 42.1 to 55.6 nm after adding anti-PYO, which demonstrates that the antibodies are becoming attached to the surface of the colloids (Fig. 2A). Subsequent steps of nanoprobe manufacturing (blocking and stabilization) did not change these parameters significantly, which suggests that antibodies are not detaching from the surface (Fig. 2A). Next, we studied whether PC1-BSA was attached to the paper matrix through the proposed spotting and drying procedure. Figure 2B shows fluorescence images obtained with a surrogate fluorescein-BSA conjugate, which show that 60% of the conjugate remains attached to the paper after rehydrating. Immunodetection experiments were then designed to demonstrate that the albumin in the conjugate is responsible for the binding to the paper. In Fig. 2C, when Ab-NPs are released from the reservoir and the paper substrate is modified with PC1-BSA, the nanoprobes yield a dose-dependent signal when the concentration of AuNPs in the reservoirs is 14.4 nM (Figure S1 shows the experiments performed for optimizing the Ab-AuNPs concentration). However, the same experiments performed with paper substrates modified with unconjugated PYO yielded lower signals that rapidly plateaued. These experiments confirm that albumin-antigen conjugates are bound to the paper, and that they yield higher immunorecognition signals compared to the direct physisorption of PYO, which justifies their integration as a competing recognition element in the biosensor. Then, we demonstrated that nanoprobes can be used for detecting PYO in a competitive immunoassay format. PC1-BSA was added up 1.52 µM PC1 to design the competitive immunoassay because it yielded the highest signal in Fig. 2C and therefore would result in the widest possible dynamic range. In Fig. 2D, it is shown that *S* signals produced by the biosensor are the highest in the absence of PYO as Ab-AuNPs can specifically recognize the immobilized PC1-BSA in the absence of PYO competition. However, adding PYO drastically decreases *S* signals (Fig. 2D), since the interaction of AuNPs with immobilized PC1-BSA is impeded by competition with the analyte (competition step in Fig. 1). Finally, in supplementary Figure S2, it is demonstrated that increasing the AuNPs size destabilizes the nanoprobes after modification with antibodies, which makes them less suitable for detecting PYO in the proposed biosensing platform (Figure S2).

**Fig. 3:**
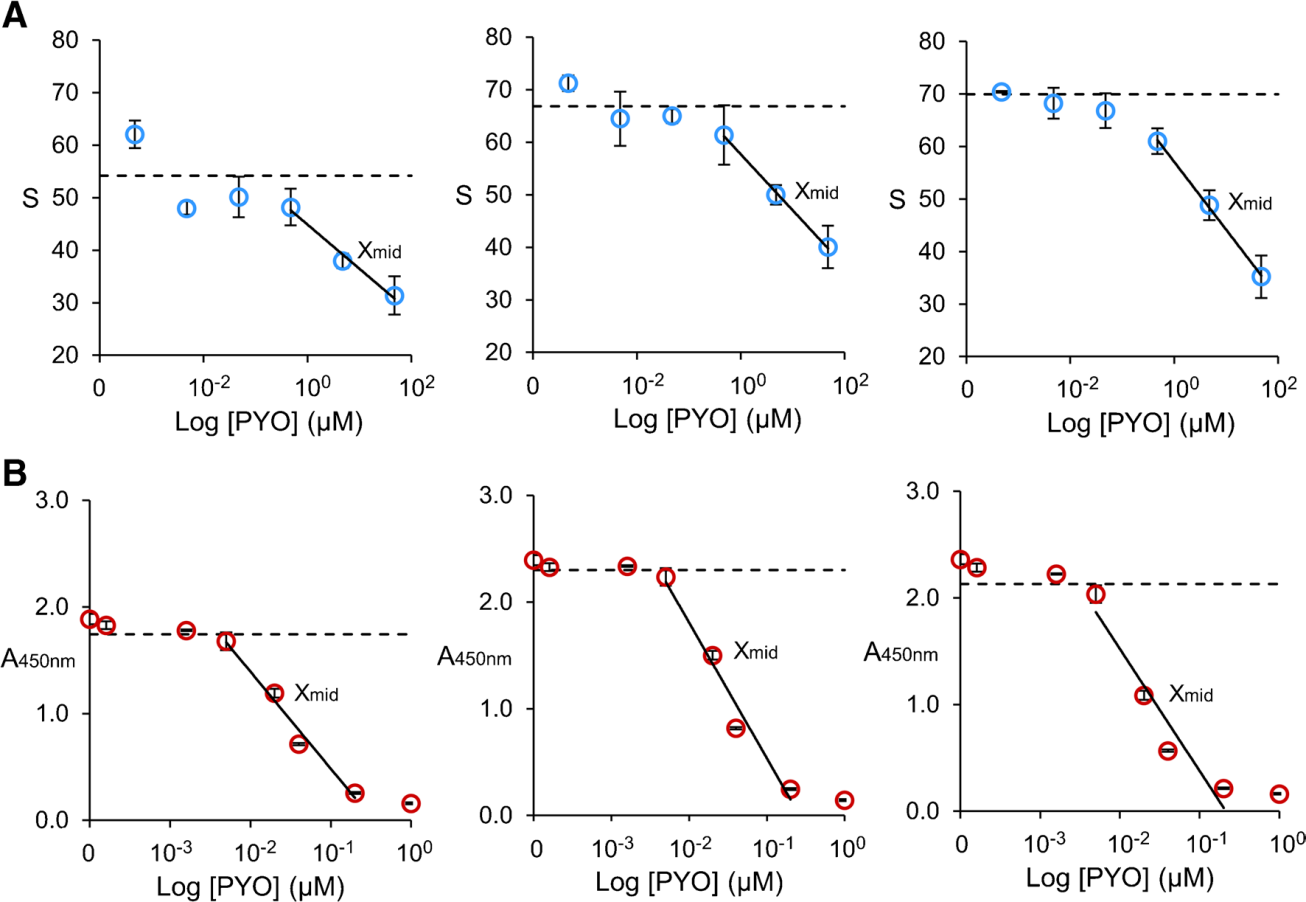
Performance of the competitive paper-based immunoassay and ELISA. Three independent linear-logarithmic calibration plots of **A** the proposed direct competitive biosensor on paper substrates and **B** the indirect competitive ELISA on microwell, for detecting pyocyanin (PYO) in PBS-BSA and PBST, respectively. PYO concentrations range from 4.76·10^−3^ to 47.6 µM in (**A**) and 1.6·10^−4^ to 1 µM in (**B**). Dotted lines represent the limit of detection (LOD = $\overline{X }$_blank_ + 3SD_blank_). Error bars are the standard deviation of 3 independent experiments. *X*_mid_ labels indicate colorimetric *S* signals in (**A**) and *A*_450_ measures in (**B**) used in the analysis of the intra- and inter-assay variability showed in Table 1

Figure 3 compares three independent calibration plots obtained with the paper biosensor and a competitive ELISA performed in microtiter plates. As expected from the competitive immunoassay format, colorimetric *S* signals yielded by the biosensor and absorbance values from ELISA decrease as the concentration of PYO increases. All the calibration plots obtained with the biosensor exhibit a linear range covering two orders of magnitude from 4.7·10^−1^ µM to 47.6 µM, with a limit of detection of 4.7·10^−3^ µM (Fig. 3A). Likewise, the calibrations of the gold standard ELISA yield linear signals in a shorter range between 5.0·10^−3^ µM and 2.0·10^−1^ µM, with a lower detection limit of 1.6·10^−3^ µM (calibration plots in Fig. 3B). Table 1 shows that the inter- and intra-assay variability is comparable between the proposed paper biosensor and the ELISA when analyzing the S signal and absorbance values yielded by the middle point within the linear range of the PYO calibration plots (X_mid_ in Fig. 3A, B, respectively). Comparing both methods, the limit of detection of ELISA is ca. 3 times lower than the one obtained with the biosensor, even though ELISA requires 2 h to be completed and a fully equipped laboratory whereas the biosensor detects PYO in 6 min without using any infrastructure. In any case, the limit of detection of both methods is much lower than the PYO levels reported in respiratory samples from patients infected by *P. aeruginosa* (> 100 µM) [28]. However, ELISA uses enzymes to amplify signals, which are labile and less suitable for in-field measurements than AuNPs. Of note, the biosensor shows a larger dynamic range. This might be associated to the fact that the 3D cellulose matrix of paper substrates allows them to immobilize a higher number of PC1-BSA molecules compared to the 2D surface of microplate wells.

**Table 1:** Intra- and inter-assay variability measured as the relative standard deviation (RSD) of the *A*_450_ and the colorimetric *S* signals produced by the competitive ELISA and the proposed paper-based biosensor in the middle point within the linear range of the PYO calibration plots (*X*_mid_ in Fig. 3A, B, respectively)

	Intra-assay RSD			Inter-assay RSD
	Cal 1	Cal 2	Cal 3	
ELISA	3.5%	0.5%	3.9%	17.1%
BIOSENSOR	3.6%	3.7%	5.8%	14.5%

**Fig. 4:**
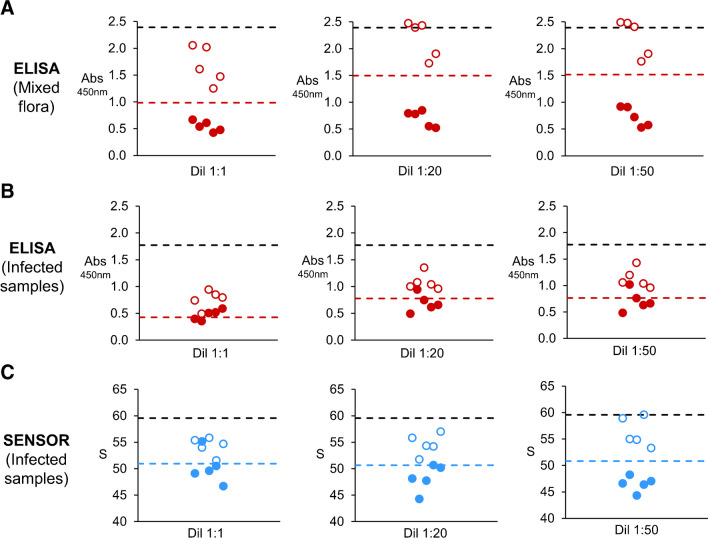
Evaluation of matrix interferences in the competitive ELISA and the biosensor for detecting sputum PYO. Absorbance at 450 nm (*A*_450_) or colorimetric *S* signal produced by liquefied samples diluted 1:1, to 1:20 or 1:50 with PBST or PBS-BSA, respectively. Dots represent the average of 3 repeated *A*_450_ or *S* measures for each sample without PYO spike (open dots), and the matched samples spiked with a constant concentration of PYO (full dots). Sputum samples containing a mixed flora analyzed with ELISA (**A**); Sputum samples from patients with a respiratory infection not caused by *P. aeruginosa* analyzed with ELISA (**B**) or with the paper biosensor (**C**). The black dotted lines represent the average of 3 replicates produced by PBST in **A** and **B** and PBS-BSA in **C** (zero with no matrix effects). The color dotted lines represent the cut-off values to differentiate non-spiked and spiked samples analyzed with ELISA (red lines) or with the paper biosensor (blue lines). Cut-off values were calculated as follows: Cut-off = $\overline{X }$_non-spiked_ – 2SD_non-spiked_

Next, we conducted a set of matrix-effect studies in order to determine the potential interferences affecting the performance of the competitive immunoassays. For this purpose, in Fig. 4 sputum samples were liquefied, diluted to different extents and spiked with a constant concentration of PYO. Non-spiked and spiked samples where then measured with ELISA and the proposed biosensor. Cut-off values to determine statistical differences between non-spiked (negative) and spiked (positive) samples were defined as two standard deviations below the mean value of non-spiked signals (color dotted lines in Fig. 4). First, we explored the differences in matrix effects originating from the presence or absence of a lung infection, since the viscosity of sputum samples and their matrix complexity increases during infections due to an enrichment in biopolymer content (e.g., mucin and DNA), the presence of lung-infiltrating leukocytes, or the higher transudation of proteins from plasma to lung secretions [29–32]. These experiments were performed with the gold standard (competitive ELISA on microtiter plates). In Fig. 4A, non-spiked samples and samples spiked with 2.4·10^−2^ µM PYO from patients with no infection, that were classified as “mixed flora” in the Microbiology Department of Son Espases Hospital, yielded clear differences when analyzed with traditional ELISA. This allowed the correct classification of all spiked samples without infection as true positive tests (full dots in Fig. 4A), that is, the samples yielded *A*_450_ values clearly below the cut-off value (red line in Fig. 4A). The best results were obtained when the samples were diluted 1:20 or 1:50 after liquefaction. However, samples with bacterial respiratory infection caused by a pathogen other than *P. aeruginosa* yielded similar results before and after being spiked, even when the samples were highly diluted (Fig. 4B). Indeed, when no dilution was done, 3 infected samples spiked with PYO yielded false negative results (full dots over the red line in left panel of Fig. 4B). Of note, the *A*_450_ vastly decreased prior to being spiked with PYO compared to the ideal experiments without matrix (black dotted line in Fig. 4B). This indicates that the matrix is blocking the interaction between the enzyme-labelled antibodies and the antigen-BSA conjugates bound to the plate. This agrees with the observation that *A*_450_ values from non-spiked samples when diluted 1:20 and 1:50 get closer to those measured in ideal experiments without matrix (black dotted lines in Fig. 4B), since this interfering blocking effect decreases with matrix dilution. However, 1 infected sample spiked with PYO continued to produce a false negative result regardless dilution (full dot over the red line in middle and right panels in Fig. 4B). These experiments show that diagnosing *P. aeruginosa* infections through the detection of sputum PYO with ELISA may lead to false results when using the proposed experimental setup, because there is not clear cut-off value separating spiked from non-spiked samples. We then checked whether paper biosensors could improve the detection of sputum PYO with the proposed sample treatment protocol. In Fig. 4C, undiluted samples without PYO yielded *S* signals that are far away from the value obtained in ideal conditions (i.e., in PBS, dotted black line). Nevertheless, only 1 spiked sample produced a false negative result (full dot over the blue line in left panel of Fig. 4C). As the dilution factor increases, the colorimetric signal *S* obtained from non-spiked samples gets closer to the results obtained in ideal experiments without matrix (black dotted line in Fig. 4C), thus suggesting a decrease in matrix effects preventing antibody-antigen interactions with substrate-bound bioconjugates. Furthermore, as the dilution factor increases, *S* signals for spiked samples move away the cut-off value, even though they all have the same final PYO concentration (2.4 µM). Since experiments with non-spiked samples show that this is not related to interactions with substrate-bound bioconjugates, these results indicate that a higher sample dilution favors interactions with free PYO in the sample. In other words, diluting the sample to a higher extend makes it easier for antibodies to interact with PYO in solution. Indeed, when the liquefied samples are diluted 1:50, there is a clear cut-off between spiked and non-spiked specimens (dotted blue lines), which proves that paper biosensor can detect sputum PYO under this condition.

**Fig. 5:**
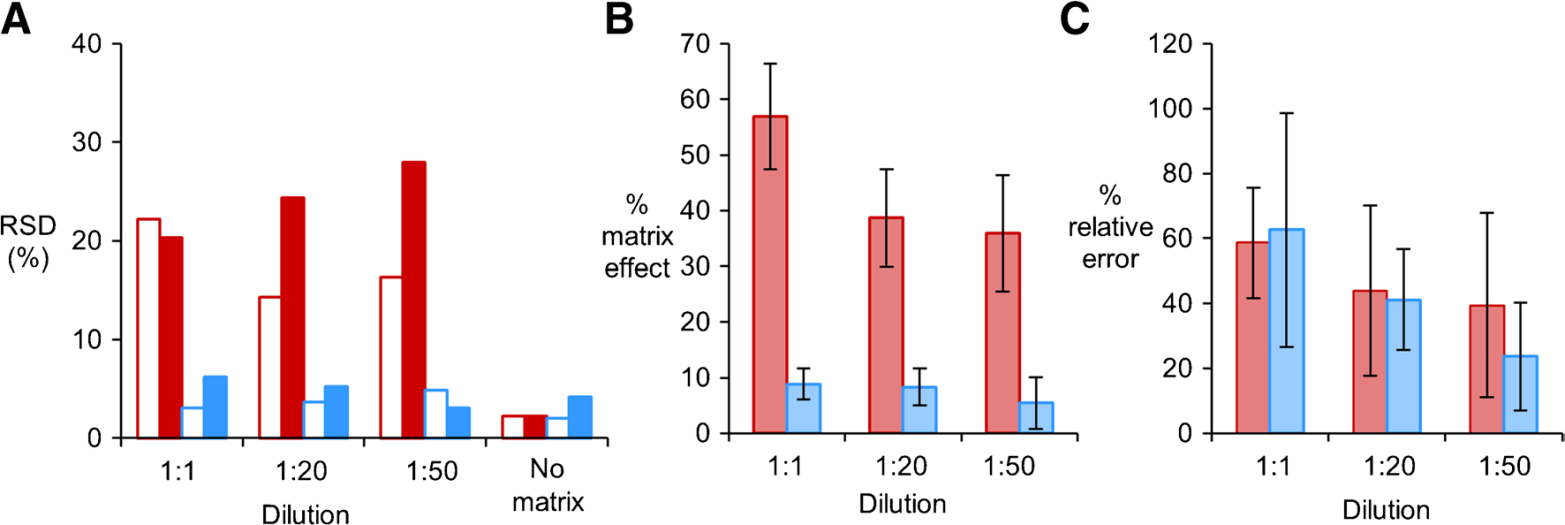
Analytical validation of competitive ELISA and the biosensor for detecting sputum PYO. **A** Inter-sample variability measured as the relative standard deviation (RSD) of *A*_450_ and colorimetric *S* signals produced by non-spiked (open bars) or spiked (full bars) sputum samples and diluted to different ratios by using the competitive ELISA (red bars) or the proposed paper-based biosensor (blue bars). RSD values of *A*_450_ and colorimetric *S* signals produced by non-spiked and spiked PBST (ELISA) or PBS-BSA (biosensor) solutions are referred as “No matrix” results. **B** Percentage of matrix effect evaluated in spiked sputum samples analyzed by the competitive ELISA (red bars) and the proposed paper-biosensor (blue bars). Error bars represent the standard deviation of the average values calculated from 3 repeated measures for each sample. **C** Accuracy of sputum PYO detection by the competitive ELISA (red bars) and the proposed paper-biosensor (blue bars), measured as the relative error of the signal decrease due to PYO spike. Error bars represent the standard deviation of the average values calculated from 3 repeated measures for each sample. In (**A**), (**B**), and (**C**) undiluted or diluted sputum samples with an infection (not caused by *P. aeruginosa*) and spiked with constant concentration of PYO were used

Finally, in Fig. 5. we evaluated the influence of sputum matrix on the reliability of PYO detection. Figure 5A shows the inter-sample variability, evaluated as the relative standard deviation (RSD) of signals yielded by ELISA and the biosensor, when the analyte was added at a constant concentration. In Fig. 5A, the RSD is less than 5% when PYO is spiked into PBS (labelled as “No matrix” in Fig. 5A). However, if PYO is spiked into sputum, the matrix has a detrimental impact on its detection, that is, the RSD increases, when using ELISA even when the sample is highly diluted (1:1 to1:50, full red bars in Fig. 5A). Indeed, the RSD is always lower when samples are measured with the paper biosensor, and mean values remain closer to the ideal situation for detecting PYO without matrix (blue bars in Fig. 5A). Next, in Fig. 5B, we evaluated the percentage of matrix effect, which indicates the % of the colorimetric signal generated by the matrix and not by the recognition of PYO. In Fig, 5B, matrix effects are higher when samples are analyzed with ELISA in all experiments, even after diluting the samples to a large extent (red bars in Fig. 5B). Conversely, samples measured with the paper biosensor never exceeded 10% of matrix effect (blue bars in Fig. 5B). Finally, we sought to evaluate the accuracy of the proposed biosensor when tested with sputum samples. Figure 5C shows the accuracy of ELISA and the biosensor, evaluated as the relative error of the signal decrease due to PYO spike. The best accuracy, that is, the lowest relative error values, was accomplished with the proposed biosensor when sputum samples are diluted 1:50 (blue bars in Fig. 5C). Nevertheless, relative error values in Fig. 5C are accompanied by large standard deviation values, which mean that in some cases, the signal decrease due to PYO spike in samples significantly differs from the one obtained in PBS. In summary, these experiments demonstrate that the paper biosensor not only reduces matrix effects but also decreases the inter-sample variability, which are all relevant parameters for diagnosing infections using sputum PYO as a biomarker. However, in terms of accuracy, the biosensor can only yield qualitative results when analyzing sputum samples from infected patients.

## Conclusions

In conclusion, we have introduced a paper-based biosensor design for the rapid detection of small molecules with competitive immunoassays. It consists of a piece of paper modified with an albumin-antigen conjugate and a paper-based reservoir containing antibody-decorated nanoparticles. After adding a drop of sample and pressing the two elements together, the antibody-nanoparticles are released from the reservoir. This generates a dose-dependent signal as the free antigen competes with albumin conjugates for the interaction with antibody-nanoparticles. When applied to the detection of PYO, the limit of detection was 4.7·10^−3^ µM, with a dynamic range between 4.7·10^−1^ µM and 47.6 µM. Moreover, we have demonstrated that the paper biosensor is advantageous for detecting PYO in sputum compared to traditional ELISA when using the proposed liquefaction method. On the one hand, there is a clear cut-off value between spiked and non-spiked samples when analyzing them with paper biosensor. This means that the paper biosensor is well suited for detecting PYO in infected sputum samples when the concentration of the analyte is higher than 2.4 µM. Although the detection of PYO in sputum is qualitative (yes/no answer), this threshold value is well below the reported concentration in infected respiratory samples (> 100 µM) [28]. Therefore, in real samples, applying the proposed 1:50 dilution should enable detecting *P. aeruginosa* infections. It should be noted that samples with no infection caused by this pathogen should always be negative, as PYO is only produced by *P. aeruginosa* cells. Furthermore, when experiments are performed with a paper biosensor, the RSD and percentage of matrix effect obtained from measuring samples from different patients are smaller compared to ELISA. This is particularly relevant for analyzing biomarkers in samples with high inter-personal matrix variability such as sputum specimens. The assay can be completed within 5 min and does not require any harsh chemicals or specialized equipment. Qualitative results could be interpreted by eye with the aid of a color chart or using a smartphone as a reader [33]. These features make our paper biosensor promising candidate for diagnosing pneumonia caused by *P. aeruginosa* through the detection of sputum PYO at the bedside.

### Supplementary Information

Below is the link to the electronic supplementary material.

Supplementary file1 (DOCX 264 KB)

## Data Availability

The data supporting the findings of this study are available from the corresponding author (A.C.) upon reasonable request.

## References

[1] Reynolds D, Kollef M (2021). The epidemiology and pathogenesis and treatment of Pseudomonas aeruginosa infections: An Update. Drugs.

[2] Hernández-Jiménez P, López-Medrano F, Fernández-Ruiz M, Silva JT, Corbella L, San-Juan R, Lizasoain M, Díaz-Regañón J, Viedma E, Aguado JM (2022) Risk factors and outcomes for multidrug resistant, *Pseudomonas aeruginosa* infection in immunocompromised patients. Antibiotics (Basel) 11(11):1459 10.3390/antibiotics11111459 PMC9686626 36358114

[3] Alonso B, Fernández-Barat L, Di Domenico EG, Marín M, Cercenado E, Merino I, de Pablos M, Muñoz P, Guembe M (2020). Characterization of the virulence of Pseudomonas aeruginosa strains causing ventilator-associated pneumonia. BMC Infect Dis.

[4] Motowski H, Ilges D, Hampton N, Kollef MH, Micek ST (2023). Determinants of mortality for ventilated hospital-acquired pneumonia and ventilator-associated pneumonia. Crit Care Explor.

[5] Marchaim D, Kaye D, Kaye KS (2019). Use of colistin in critically Ill patients. Adv Exp Med Biol.

[6] Jochumsen N, Marvig RL, Damkiær S, Jensen RL, Paulander W, Molin S, Jelsbak L, Folkesson A (2016). The evolution of antimicrobial peptide resistance in Pseudomonas aeruginosa is shaped by strong epistatic interactions. Nat Commun.

[7] Tumbarello M, De Pascale G, Trecarichi EM, Spanu T, Antonicelli F, Maviglia R, Pennisi MA, Bello G, Antonelli M (2013). Clinical outcomes of Pseudomonas aeruginosa pneumonia in intensive care unit patients. Intensive Care Med.

[8] Zakhour J, Sharara SL, Hindy JR, Haddad SF, Kanj SS (2022) Antimicrobial treatment of *Pseudomonas aeruginosa* severe sepsis. Antibiotics (Basel) 11(10):1432 10.3390/antibiotics11101432 PMC9598900 36290092

[9] Rada B, Leto TL (2013). Pyocyanin effects on respiratory epithelium: relevance in Pseudomonas aeruginosa airway infections. Trends Microbiol.

[10] Ferreira R, Pãixao TR, Torres MDT, de Araujo WR (2020). Simple and inexpensive electrochemical paper-based analytical device for sensitive detection of Pseudomonas aeruginosa. Sens Actuators, B Chem.

[11] Alatraktchi FA, Noori JS, Tanev GP, Mortensen J, Dimaki M, Johansen HK, Madsen J, Molin S, Svendsen WE (2018). Paper-based sensors for rapid detection of virulence factor produced by *Pseudomonas aeruginosa*. PLoS ONE.

[12] Alatraktchi FA, Dimaki M, Støvring N, Johansen HK, Molin S, Svendsen WE (2020). Nanograss sensor for selective detection of Pseudomonas aeruginosa by pyocyanin identification in airway samples. Anal Biochem.

[13] Rashid JIA, Kannan V, Ahmad MH, Mon AA, Taufik S, Miskon A, Ong KK, Yusof NA (2021). An electrochemical sensor based on gold nanoparticles-functionalized reduced graphene oxide screen printed electrode for the detection of pyocyanin biomarker in *Pseudomonas aeruginosa* infection. Mater Sci Eng C Mater Biol Appl.

[14] Thulasinathan B, Murugan SDS, Panda SK, Veerapandian M, Manickam P (2023). DNA-functionalized carbon quantum dots for electrochemical detection of pyocyanin: a quorum sensing molecule in *Pseudomonas aeruginosa*. Biosens Bioelectron.

[15] Pastells C, Pascual N, Sanchez-Baeza F, Marco MP (2016). Immunochemical determination of pyocyanin and 1-hydroxyphenazine as potential biomarkers of *Pseudomonas aeruginosa* Infections. Anal Chem.

[16] Rodriguez-Urretavizcaya B, Pascual N, Pastells C, Martin-Gomez MT, Vilaplana L, Marco MP (2021). Diagnosis and stratification of *Pseudomonas aeruginosa* infected patients by immunochemical quantitative determination of pyocyanin from clinical bacterial isolates. Front Cell Infect Microbiol.

[17] Wu X, Chen J, Li X, Zhao Y, Zughaier SM (2014). Culture-free diagnostics of Pseudomonas aeruginosa infection by silver nanorod array based SERS from clinical sputum samples. Nanomedicine.

[18] Žukovskaja O, Agafilushkina S, Sivakov V, Weber K, Cialla-May D, Osminkina L, Popp J (2019). Rapid detection of the bacterial biomarker pyocyanin in artificial sputum using a SERS-active silicon nanowire matrix covered by bimetallic noble metal nanoparticles. Talanta.

[19] Žukovskaja O, Jahn IJ, Weber K, Cialla-May D, Popp J (2017) Detection of Pseudomonas aeruginosa metabolite pyocyanin in water and saliva by employing the SERS technique. Sensors (Basel) 17(8):1704 10.3390/s17081704 PMC5580190 28757555

[20] Frigoli M, Lowdon JW, Caldara M, Arreguin-Campos R, Sewall J, Cleij TJ, Diliën H, Eersels K, van Grinsven B (2023). Thermal pyocyanin sensor based on molecularly imprinted polymers for the indirect detection of *Pseudomonas aeruginosa*. ACS Sens.

[21] Saraswathy Veena V, Sara George P, Jayasree K, Sujathan K (2015). Comparative analysis of cell morphology in sputum samples homogenized with dithiothreitol, N-acetyl-L cysteine, Cytorich^(R)^ red preservative and in cellblock preparations to enhance the sensitivity of sputum cytology for the diagnosis of lung cancer. Diagn Cytopathol.

[22] Clemente A, Alba-Patiño A, Rojo-Molinero E, Russell SM, Borges M, Oliver A, de la Rica R (2020). Rapid detection of *Pseudomonas aeruginosa* biofilms via enzymatic liquefaction of respiratory samples. ACS Sens.

[23] Clemente A, Alba-Patiño A, Santopolo G, Barón E, Rojo-Molinero E, Oliver A, Pérez-Bárcena J, Merino de Cos P, Aranda M, Del Castillo A, Socias A, Borges M, de la Rica R (2021). Optimized detection of lung IL-6 *via* enzymatic liquefaction of low respiratory tract samples: application for managing ventilated patients. Analyst.

[24] Santopolo G, Clemente A, Rojo-Molinero E, Fernández S, Álvarez MC, Oliver A, de la Rica R (2022). Improved cytometric analysis of untouched lung leukocytes by enzymatic liquefaction of sputum samples. Biol Proced Online.

[25] Kimling J, Maier M, Okenve B, Kotaidis V, Ballot H, Plech A (2006). Turkevich method for gold nanoparticle synthesis revisited. J Phys Chem B.

[26] Lou S, Ye JY, Li KQ, Wu A (2012). A gold nanoparticle based immunochromatographic assay: the influence of nanoparticulate size. Analyst.

[27] Vaquer A, Adrover-Jaume C, Clemente A, Iglesias A, López M, Martínez R, Roig IM, Cosío BG, de la Rica R (2023). Immunosensors made of polymer-infused porous paper for the non-invasive detection of airways cytokines trapped by porous face masks. Sens Actuators, B Chem.

[28] Hall S, McDermott C, Anoopkumar-Dukie S, McFarland A, Forbes A, Perkins A, Davey A, Chess-Williams R, Kiefel M, Arora D, Grant G (2016). Cellular effects of pyocyanin, a secreted virulence factor of *Pseudomonas aeruginosa*. Toxins.

[29] Buzid A, Shang F, Reen FJ, Muimhneacháin E, Clarke SL, Zhou L, Luong JH, O'Gara F, McGlacken GP, Glennon JD (2016). Molecular signature of *Pseudomonas aeruginosa* with simultaneous nanomolar detection of quorum sensing signaling molecules at a boron-doped diamond electrode. Sci Rep.

[30] Guan WJ, Huang Y, Chen CL, Yuan JJ, Li HM, Gao YH, Chen RC, Zhong NS (2018). Sputum purulence-associated microbial community compositions in adults with bronchiectasis. J Thorac Dis.

[31] Vogel L, Schoonbrood D, Geluk F, Hoek F, Bresser P, Out T, Jansen H, Dankert J, van Alphen L (1997). Iron-binding proteins in sputum of chronic bronchitis patients with *Haemophilus influenzae* infections. Eur Respir J.

[32] Yuan S, Hollinger M, Lachowicz-Scroggins ME, Kerr SC, Dunican EM, Daniel BM, Ghosh S, Erzurum SC, Willard B, Hazen SL, Huang X, Carrington SD, Oscarson S, Fahy JV (2015) Oxidation increases mucin polymer cross-links to stiffen airway mucus gels. Sci Transl Med 7(276):276ra27 10.1126/scitranslmed.3010525 PMC4403633 25717100

[33] Alba-Patiño A, Russell SM, Borges M, Pazos-Pérez N, Álvarez-Puebla RA, de la Rica R (2020). Nanoparticle-based mobile biosensors for the rapid detection of sepsis biomarkers in whole blood. Nanoscale Adv.

