# Supplementary material for: A paper biosensor for overcoming matrix effects interfering with the detection of sputum pyocyanin with competitive immunoassays
Source: Mikrochim Acta. 2023 Oct 16;190(11):441. doi: 10.1007/s00604-023-06017-1 (PMC10579119; doi:10.1007/s00604-023-06017-1)
Supplement: Supplementary file 1 — Supplementary file1 (DOCX 264 KB) [file 604_2023_6017_MOESM1_ESM.docx]

**Supporting information for:**

**A Paper Biosensor for Overcoming Matrix Effects Interfering with the Detection of Sputum Pyocyanin with Competitive Immunoassays**

Cristina Adrover-Jaume,^1,2^ Antonio Clemente,^1,2,3,^* Bárbara Rodríguez-Urretavizcaya,^4,5^ Lluïsa Vilaplana,^4,5^  M. Pilar Marco,^4,5^ Estrella Rojo-Molinero,^3,6^ Antonio Oliver,^3,6^ and Roberto de la Rica.^1,3^

^1^Multidisciplinary Sepsis Group, Hospital Universitario Son Espases, Health Research Institute of Balearic Islands (IdISBa), Palma de Mallorca, Spain

^2^Department of Chemistry, University of the Balearic Islands, Palma de Mallorca, Spain.

^3^CIBER de Enfermedades Infecciosas (CIBERINFEC), Instituto de Salud Carlos III Madrid, Spain.

^4^Nanobiotechnology for Diagnostics (Nb4D), Department of Surfactants and Nanobiotechnology, Institute for Advanced Chemistry of Catalonia (IQAC), Barcelona, Spain.

^5^CIBER de Bioingeniería, Biomateriales y Nanomedicina (CIBER-BBN), Barcelona, Spain.

^6^Microbiology Department, Hospital Universitario Son Espases, Health Research Institute of Balearic Islands (IdISBa), Palma de Mallorca, Spain.

*Corresponding authors: antonio.clemente@ssib.es

**LIST OF CONTENTS**

**Figure S1.** Impact of 20 nm Ab-AuNPs concentration on the colorimetric S signals obtained with PYO immunosensors.

**Figure S2.** PYO detection by competitive immunoassays with 40 nm Ab-AuNPs.

**Table S1.** Detailed comparison between this work and other reported methods for the rapid detection of *Pseudomonas* infections.

**Table S2.** Bacteriological culture findings in the sputum samples included in the study.

**Table S3.** Average size of AuNPs in TEM images.

**S1. Optimization of 20 nm Ab-AuNPs**

Reservoirs with different concentration of 20 nm Ab-AuNPs (3.6, 7.2, 14.4 and 28.9 nM) were prepared as explained in the materials and methods section of the main text. Then, 10 µl PC1-BSA (at 1.52 µM PC1) or PBS was added to a piece of paper and dried at RT for 10 minutes. Next, 0.5 mL PBS-BSA was added to the paper substrate and Ab-AuNPs were transferred by pressing the reservoir against the detection substrate with a clamp for 5 minutes. Finally, the paper substrate with the Ab-AuNPs bound to PC1-BSA was washed 6 times with PBST (total volume 1 mL) and the colorimetric S signal was evaluated by densitometry.

Figure S1 shows that 14.4 nM Ab-AuNPs yields the best signal-to-noise increase (ΔS) with the lower signal variability.


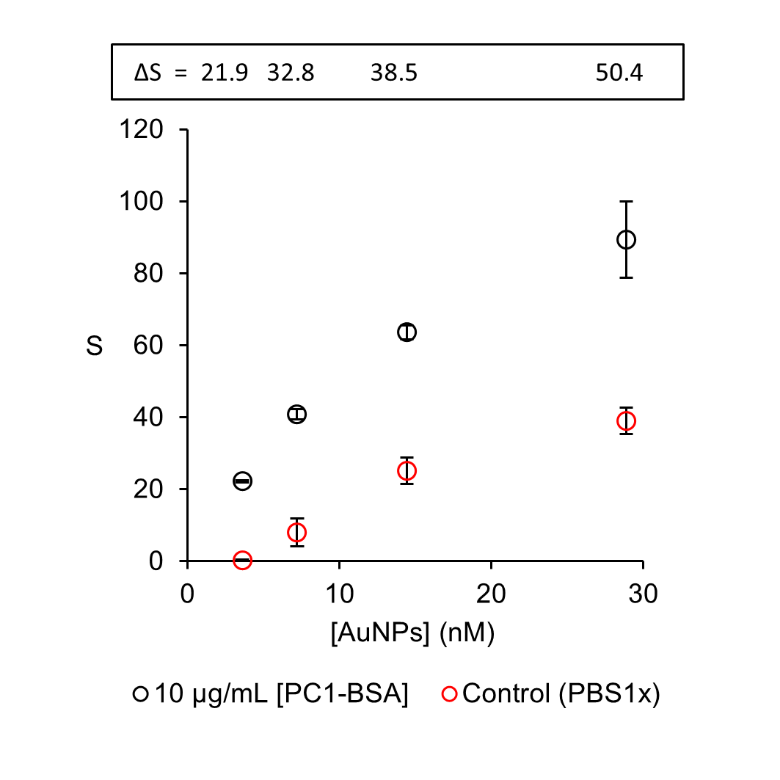


**Figure S1. Impact of Ab-AuNPs concentration on the colorimetric S signals obtained with PYO immunosensors.** Colorimetric S signals yielded by increasing concentrations of Ab-AuNPs transferred to paper substrates modified with PC1-BSA bioconjugate (black dots) or PBS (red dots). Error bars are the SD of 3 independent measurements. ΔS = S_PC1-BSA_ - S_PBS._

**S2. Competitive immunoassays with 40 nm Ab-AuNPs.**

Competitive immunoassays with 40 nm Ab-AuNPs were done in order to evaluate the impact of AuNPs size on the biosensor performance. First, 40 nm AuNPs were synthesized using the Turkevich method [25]. In brief, 0.75 mM trisodium citrate was added to a boiling aqueous solution of 0.5 mM gold chloride for 15 min while vigorously stirring (final volume 250 mL). The resulting AuNPs were let to cool down at room temperature. Then, 40 nm AuNPs were modified with antibodies against PYO following the physical adsorption protocol shown in the Experimental Section of the main text. Finally, 40 nm Ab-AuNPs were used in paper-based direct competitive assays for detecting PYO, following the same protocol for 20 nm Ab-AuNPs reported in the Experimental Section of the main text.

In Figure S2A the LSPR peak after their synthesis (black spectrum) is located at 530 nm, as expected for 40 nm AuNPs. Vis-NIR spectroscopy after subsequent steps of nanoprobes manufacturing (addition of antibodies, BSA and sucrose) demonstrates that 40 nm AuNPs colloids become consequently destabilized, since the LSPR red-shifts and widens which indicates colloidal aggregation (Figure S2A). Accordingly, in Figure S2B it is demonstrated that 40 nm Ab-AuNPs are less suitable for detecting PYO with our competitive biosensor, given that the biosensor yields similar signals in the absence and in the presence of PYO.


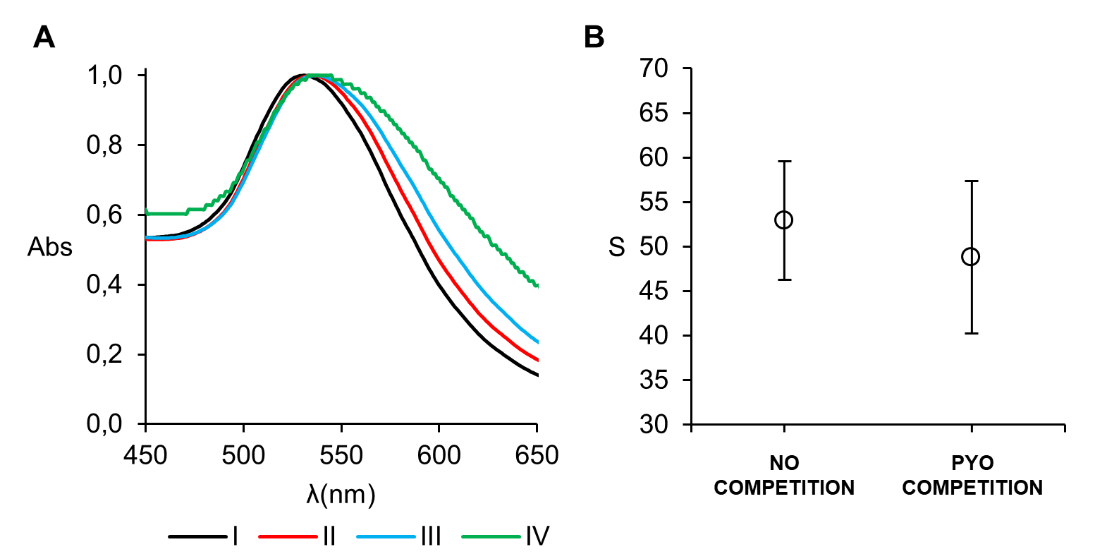


**Figure S2. PYO detection by competitive immunoassays with 40 nm Ab-AuNPs. (A)** Vis-NIR spectroscopy of 40 nm gold nanoparticles (AuNPs) after the synthesis (I, black) and after each step of the functionalization protocol; addition of antibodies (II, red), stabilization with BSA (III, blue), and addition of sucrose (IV, green). **(B)** Colorimetric S signals produced by Ab-AuNPs in a direct paper-based competitive immunoassay in the absence of PYO (no competition) and with 47.6 μM PYO in PBS-BSA (PYO competition).

**Table S1. Detailed comparison between this work and other reported methods for the rapid detection of *Pseudomonas* infections.**

| **Ref.** | **Analyte** | **Detection platform** | **Sensor type** | **Respiratory samples** | **Analysis time** | **Detection equipment** | **LOD** | **LDR** |
| --- | --- | --- | --- | --- | --- | --- | --- | --- |
| **Our method** | Pyocyanin | Paper | Optical | Yes | 5 min | Mobile app [33] | 4.7 · 10^-3^ µM | 4.7 · 10^-3^ – 47.6 µM |
| [10] | Pyocyanin | Paper | Electrochemical | No | 10 min | Potentiostat | 10 nM | 0.05 – 1 µM |
| [11] | Pyocyanin | Paper | Electrochemical | No | - | Potentiostat | 95 nM | 1 – 40 µM |
| [12] | Pyocyanin | Gold coated nanograss | Electrochemical | Yes | 1 min | Potentiostat | 172 nm | 0.313 – 25 µM |
| [14] | Pyocyanin | Gold | Electrochemical | No | - | Potentiostat | 2 µM | 2 – 100 µM |
| [20] | Pyocyanin | Molecularly imprinted polymers (MIPs) | Thermal | Yes | - | Thermal transducer | 0.347 ± 0.027 µM | 0-30 µM |
| [22] | *Pseudomonas aeruginosa* | Paper | Optical | Yes | 8 min | Mobile app | 10^5^ cells·mL^-1^ | 10^4^ – 10^9^ cells·mL^-1^ |
| Webster, T.A.; Sismaet, H.J.; Conte, J.L.; Chan, I.; Ping, J.; Goluch, E.D. Electrochemical detection of  Pseudomonas aeruginosa in human fluid samples via pyocyanin. Biosens. Bioelectron. **2014**, 60, 265–270. | Pyocyanin | Carbon | Electrochemical | Yes | < 5 min | Potentiostat | 0.13 – 1.81 µM | 1 – 100 µM |
| L.H. Kim, H.W. Yu, Y.H. Kim, I.S. Kim, A. Jang, Potential of fluorophore labeled aptamers for Pseudomonas aeruginosa detection in drinking water, Journal of the Korean Society for Applied Biological Chemistry 56 (2013) 165e171, | *Pseudomonas aeruginosa* | Solution | Optical | No | 30 min | Fluorescence spectrophotometer | 5.07 cells·mL^-1^ | 5.64 – 10^2^ cells·mL^-1^ |
| Z. Zhong, R. Gao, Q. Chen, L. Jia, Dual-aptamers labeled polydopamine-polyethyleneimine copolymer dots assisted engineering a fluorescence biosensor for sensitive detection of Pseudomonas aeruginosa in food samples, Spectrochim. Acta Mol. Biomol. Spectrosc. 224 (2020) 117417 | *Pseudomonas aeruginosa* | Solution | Optical | No | 45 min | Fluorescence spectrophotometer | 1 cells·mL^-1^ | 10^1^ – 10^7^ cells·mL^-1^ |

**Table S2.** **Bacteriological culture findings in the sputum samples included in the study.**

| **Sample ID** | **Culture Test** |
| --- | --- |
| Sample 1 | MF |
| Sample 2 | MF |
| Sample 3 | MF |
| Sample 4 | MF |
| Sample 5 | MF |
| Sample 6 | *Staphylococcus aureus* |
| Sample 7 | *Proteus mirabilis* |
| Sample 8 | *Staphylococcus aureus* |
| Sample 9 | *Moraxella catarrhalis* |
| Sample 10 | *Serratia marcescens* |

**MF:** mixed flora

**Table S3.** **Average size of AuNPs in TEM images.**

Horizontal and vertical diameters of AuNPs were manually measured by using the plot profile function of ImageJ in 50 particles imaged in TEM pictures.

| **AuNP** | **Pixels** | | **nm** | | **Mean (nm)** |
| --- | --- | --- | --- | --- | --- |
|  | **Horitzontal** | **Vertical** | **Horitzontal** | **Vertical** |  |
| 1 | 114,02 | 116,02 | 19,86 | 20,21 | 20,04 |
| 2 | 138,00 | 140,23 | 24,04 | 24,43 | 24,24 |
| 3 | 147,00 | 154,00 | 25,61 | 26,83 | 26,22 |
| 4 | 169,00 | 137,00 | 29,44 | 23,87 | 26,65 |
| 5 | 136,00 | 144,01 | 23,69 | 25,09 | 24,39 |
| 6 | 135,00 | 148,00 | 23,52 | 25,78 | 24,65 |
| 7 | 105,00 | 113,11 | 18,29 | 19,70 | 19,00 |
| 8 | 131,00 | 137,06 | 22,82 | 23,88 | 23,35 |
| 9 | 120,00 | 112,02 | 20,91 | 19,51 | 20,21 |
| 10 | 117,07 | 111,00 | 20,39 | 19,34 | 19,87 |
| 11 | 148,03 | 163,01 | 25,79 | 28,40 | 27,09 |
| 12 | 142,00 | 129,00 | 24,74 | 22,47 | 23,61 |
| 13 | 123,00 | 112,00 | 21,43 | 19,51 | 20,47 |
| 14 | 100,00 | 99,00 | 17,42 | 17,25 | 17,33 |
| 15 | 101,00 | 90,00 | 17,60 | 15,68 | 16,64 |
| 16 | 170,00 | 136,00 | 29,62 | 23,69 | 26,65 |
| 17 | 140,00 | 151,00 | 24,39 | 26,31 | 25,35 |
| 18 | 134,00 | 133,00 | 23,34 | 23,17 | 23,26 |
| 19 | 114,00 | 115,00 | 19,86 | 20,03 | 19,95 |
| 20 | 108,00 | 115,00 | 18,81 | 20,03 | 19,42 |
| 21 | 110,00 | 135,00 | 19,16 | 23,52 | 21,34 |
| 22 | 107,00 | 110,00 | 18,64 | 19,16 | 18,90 |
| 23 | 108,00 | 111,00 | 18,81 | 19,34 | 19,08 |
| 24 | 121,00 | 122,00 | 21,08 | 21,25 | 21,17 |
| 25 | 141,00 | 133,00 | 24,56 | 23,17 | 23,87 |
| 26 | 125,00 | 139,00 | 21,78 | 24,22 | 23,00 |
| 27 | 128,00 | 120,00 | 22,30 | 20,91 | 21,60 |
| 28 | 137,00 | 141,00 | 23,87 | 24,56 | 24,22 |
| 29 | 113,00 | 108,00 | 19,69 | 18,81 | 19,25 |
| 30 | 137,00 | 123,00 | 23,87 | 21,43 | 22,65 |
| 31 | 118,00 | 114,00 | 20,56 | 19,86 | 20,21 |
| 32 | 116,00 | 118,00 | 20,21 | 20,56 | 20,38 |
| 33 | 99,00 | 107,00 | 17,25 | 18,64 | 17,94 |
| 34 | 97,00 | 103,00 | 16,90 | 17,94 | 17,42 |
| 35 | 104,00 | 98,00 | 18,12 | 17,07 | 17,60 |
| 36 | 96,00 | 116,00 | 16,72 | 20,21 | 18,47 |
| 37 | 94,00 | 108,00 | 16,38 | 18,81 | 17,60 |
| 38 | 129,00 | 140,00 | 22,47 | 24,39 | 23,43 |
| 39 | 94,00 | 108,00 | 16,38 | 18,81 | 17,60 |
| 40 | 121,00 | 122,00 | 21,08 | 21,25 | 21,17 |
| 41 | 118,00 | 128,00 | 20,56 | 22,30 | 21,43 |
| 42 | 109,00 | 108,00 | 18,99 | 18,81 | 18,90 |
| 43 | 104,00 | 108,00 | 18,12 | 18,81 | 18,47 |
| 44 | 130,00 | 113,00 | 22,65 | 19,69 | 21,17 |
| 45 | 112,00 | 120,00 | 19,51 | 20,91 | 20,21 |
| 46 | 107,00 | 112,00 | 18,64 | 19,51 | 19,08 |
| 47 | 120,00 | 113,00 | 20,91 | 19,69 | 20,30 |
| 48 | 119,00 | 130,00 | 20,73 | 22,65 | 21,69 |
| 49 | 133,00 | 135,00 | 23,17 | 23,52 | 23,34 |
| 50 | 139,00 | 137,00 | 24,22 | 23,87 | 24,04 |
|  |  | **Mean=** | 21,18 | 21,38 | 21,28 |
|  |  | **SD=** | 3,11 | 2,78 | 2,78 |
|  |  | **RSD (%)=** | 15% | 13% | 13% |

**Note:** The 50 nm scale bar represented in TEM images corresponds to 287.01 pixels.
